# Portable Skin Analyzers with Simultaneous Measurements of Transepidermal Water Loss, Skin Conductance and Skin Hardness

**DOI:** 10.3390/s19183857

**Published:** 2019-09-06

**Authors:** Daniel (Jai Kyoung) Sim, Sung Mok Kim, Steve S. Kim, Il Doh

**Affiliations:** 1 Center for Medical Convergence Metrology, Korea Research Institute of Standards and Science (KRISS), Daejeon 34113, Korea (D.S.) (S.M.K.); 2 711th Human Performance Wing, Air Force Research Laboratory (AFRL), Wright-Patterson Air Force Base, Dayton, OH 45433, USA; 3 Department of Medical Physics, University of Science and Technology (UST), Daejeon 34113, Korea

**Keywords:** pen-type device, multimodal skin analysis, transepidermal water loss (TEWL), skin conductance, skin hardness

## Abstract

Simultaneous measurement of skin physiological and physical properties are important for the diagnosis of skin diseases and monitoring of human performance, since it provides more comprehensive understanding on the skin conditions. Current skin analysis devices, however, require each of probes and unique protocols for the measurement of individual skin properties, resulting in inconvenience and increase of measurement uncertainty. This paper presents a pen-type skin analyzing device capable tomeasure three key skin properties at the same time: transepidermal water loss (TEWL), skin conductance, and skin hardness. It uses a single truncated hollow cone (THC) probe integrated with a humidity sensor, paired electrodes, and a load cell for the multimodal assessment of the skin properties. The present device measured TEWL with a sensitivity of 0.0068 (%/s)/(g/m^2^/h) and a linearity of 99.63%, conductance with a sensitivity of 1.02 µS/µS and a linearity of 99.36%, and hardness with a sensitivity of 0.98 Shore 00/Shore 00 and a linearity of 99.85%, within the appropriate ranges for the human skin. The present pen-type device has a high potential for the skin health diagnosis as well as the human performance monitoring applications.

## 1. Introduction

Minimally or non-invasive measurements of skin properties have been widely utilized not only for the diagnosis of skin diseases [1] such as atopic dermatitis and systemic sclerosis but also for the skin care in cosmetic fields [2]. There are a number of skin properties that reflect physiological or physical conditions of the skin. Among them, transepidermal water loss (TEWL), skin conductance, and skin hardness are key skin properties for the assessments of both the skin health and the human performance in clinics. More specifically, TEWL [3,4,5,6] and skin conductance [7,8,9] reflect the epidermal barrier function to keep the moisture in the skin and protect skin elements from contamination [10]. The TEWL is a measure of water loss from a skin surface, and the skin conductance is a measure of skin moisture. Low TEWL and high skin conductance are the indicators of healthy barrier function condition. Therefore, measurement of TEWL and skin conductance help understand epidermis status. TEWL and skin conductance are also related to human physiological status. Higher level of TEWL can be considered as “sweat rate” which is a key indicator of the human thermal status [11,12]. Skin conductance is highly related to the psychological stress status [13]. It is reported that the time-dependent conductance changes at palm and volar wrist indicate positive relation with chronic/acute stress statuses [14]. Meanwhile, skin hardness [15,16,17] reflects the skin physical condition related to collagens and elastic fibers [18]. These skin elements are in charge of protecting skin physiological elements (such as nerve endings, sweat glands, and blood vessels [19,20]) from external physical affects. A moderate level of skin hardness indicates healthy skin physical condition. For example, too high of a level of skin hardness indicates a high possibility of systemic sclerosis [16]. Skin hardness is also related to the arrector pili muscles [21]. Arrector pili muscles (also known as goose bumps) are connected to the hair follicles, having close relation with emotional feelings [22] such as cold and fear. The human physical signals transmitted through skin such as the heart sound, blood pressure, and photoplethysmogram (PPG), are also highly correlated with the hardness of the skin. In this regard, the three skin properties of TEWL, skin conductance, and skin hardness are keys to understanding the skin health status. In addition, the three skin properties can be used for the human performance monitoring, by implementing wearable electronics technologies [23,24,25].

The more skin properties we measure, the more comprehensive and in-depth understanding of the skin we can obtain than the single property based analysis. Therefore, multimodal skin analysis tools have been in demand. Previously, Grankande, et. al. [26] utilized a series of multiple probes and procedures only for single property in order to measure multiple skin properties. Using multiple probes and procedures leaded to the inconvenience as well as the increase of measurement uncertainty. Integrated single device structure, for example, a dual-modal device [27] has been recently developed for the simultaneous measurements of TEWL and skin conductance. It has a single skin contact pad composed of a chamber for measuring TEWL, and a pair of electrodes for measuring skin conductance. However, this device only accounts skin barrier functions condition, lacking the understanding of another skin physical condition. We previously reported “truncated hollow cone” (THC) probe [28] that enables to assess both barrier function (TEWL) and skin physical condition (skin hardness). The THC probe was designed with a unique structure where the upper region is cylindrical chamber for the TEWL measurement, and the lower half is narrowed for the effective hardness measurement. However, since this previous method was based on mechanical active actuation of THC probe, it required high power and an extra footprint from a bulky cylindrical supporting body.

In this paper, we present a pen-type multimodal skin analysis device capable of measuring TEWL, skin conductance, and skin hardness, simultaneously. The present pen-type device modifies the previous THC probe-based device by replacing the active actuation with a passive spring element and force sensor, which makes it possible to simplify embody into compact pen-type structure. In addition, since the new design enables the THC to contact the skin continuously, skin conductance can be obtained during the measurement by implanting the paired electrodes at the THC tip aperture. 

## 2. Experimental

### 2.1. Working Principle 

**Figure 1:**
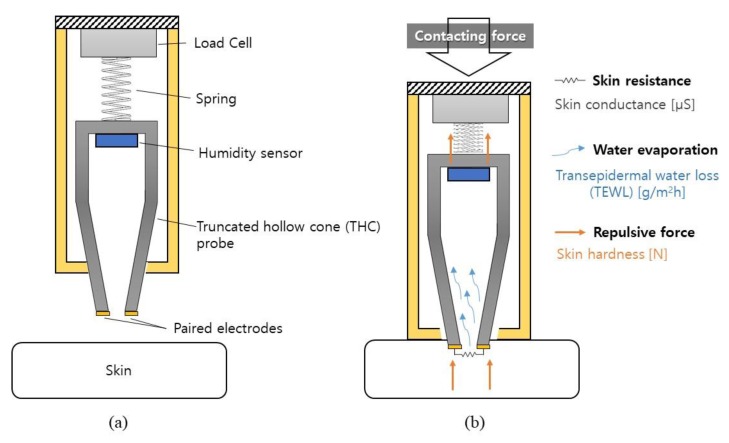
Working principle of the proposed device for assessing the skin conductance, transepidermal water loss (TEWL), and skin hardness, composed of a truncated cone probe, paired electrodes, a humidity sensor, a spring, and a load cell: (**a**) before skin contact; (**b**) after skin contact.

Figure 1 shows the schematics of the pen-type THC device that is composed of a main body, a THC probe integrated with a humidity sensor and a pair of electrodes, a mechanical coiled spring, and a load cell (Figure 1a). The THC probe is to measure TEWL, skin conductance, and skin hardness simultaneously. Figure 1b shows the overall measurement process when the THC probe is in contact with the skin. The inner chamber of the THC probe is to detect the water evaporated from the skin for TEWL measurement, where the humidity sensor is located at the top. The paired electrodes placed at the THC aperture measure the skin resistance upon the contact. The spring and the load cell attached to the THC probe are used for skin hardness measurement. Main body provides a guide for the vertical movement of the THC probe.

TEWL is derived from the pen-type device by using the Closed Chamber principle (CCP) [4]. In CCP, the TEWL is calculated from the rate of relative humidity change (Δ relative humidity/Δ time), while water evaporated from skin is being collected inside the closed chamber of THC probe. TEWL calculation considered the linear part of the humidity slope to get repeatable and stable results.

To measure skin conductance, the pen-type device uses the bipolar recording method [14,29] which is based on Ohm’s law. The detection principle is to use a paired electrodes placed on the skin and it is served as the two terminals of one resistance [29]. Constant current is applied to the paired electrodes when the probe is in contact with a skin. Applied current flows through the skin surface and a voltage drop between two electrodes is measured, and the constant current divided by the measured voltage indicates a skin conductance. 

To measure skin hardness, the device uses the Durometer principle [15,16]. It considers the depth of an indentation in the skin created by a given force on an indenter, in this case, a truncated cone probe. This depth is dependent on the hardness of the skin. Skin hardness is measured from the skin repulsive force upon exposure to the tactical force by a truncated probe [30]. The skin’s mechanical hardness is related to the measured probe’s repulsive force at the load cell. Hard skin has higher resistance to a probe indentation than soft one, thus providing stronger repulsive force.

### 2.2. Design and Implementation

**Figure 2:**
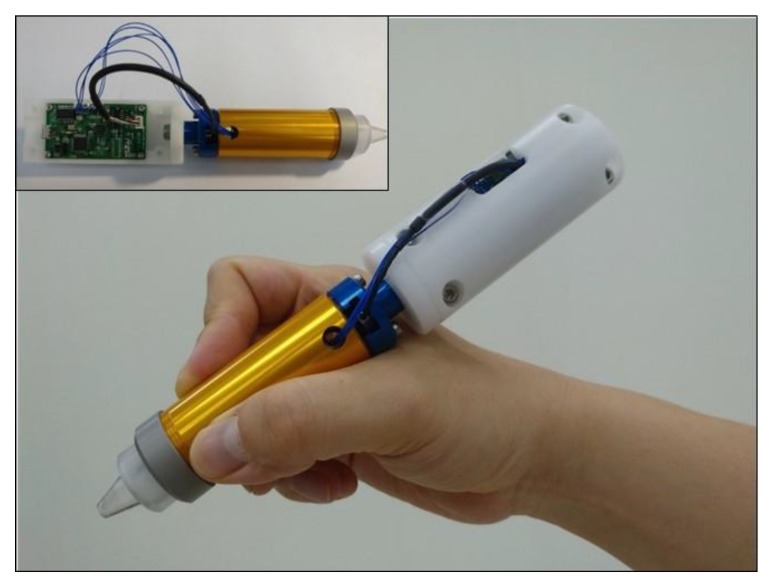
Fabricated pen-type multimodal device.

The device is designed to have a hand-held pen shape for the user-friendly ergonomic feature and portability. Total dimensions of the pen-type device are 27 mm (diameter) × 100 mm (length, upper PCB part is not included). The dimensions of the THC probe have an entrance hole diameter of 3 mm and a maximum indentation distance of 12 mm. The humidity sensor inside the THC probe is analog type sensor IC (SHT31-ARP, Sensirion, Stäfa, Switzerland) with fully calibrated, linearized, and temperature compensated output with the accuracy of ±2% in relative humidity. The two electrodes for the conductance measurement are the pair of mini-pins attached at the edge of the THC probe entrance. The load cell for the hardness measurement is a coin-shaped one (UNCRS-10N, Unipulse, Tokyo, Japan) with a diameter of 12 mm and a thickness of 4 mm. It measures the force up to 10 N with a nonlinearity of 1.0%. A mechanical spring has wire diameter of 1.7 mm, overall diameter of 8.3 mm and length of 50 mm. The PCB supplies power source to all sensors, and delivers their signals to a personal computer that displays measurement results in real-time. Customized LabVIEW (National Instruments, Austin, TX, USA) based program is developed to process and visualize signals from the sensor elements. Measured data is transmitted by USB cable connected to PCB. USB cable is also used to supply electronic power to PCB. Figure 2 shows the fabricated pen-type device integrated with PCB.

### 2.3. Calibration of the Measurements

Before the skin measurement, the present devices are needed to be calibrated. For TEWL measurement, Wet-cup method [31,32,33] has been used. Briefly, the wet-cup method uses a water-filled petri-dish covered by a semi-permeable membrane. The wet-cup provides the constant water-emitting surface and their rate can be controlled by the water temperature. The rate of evaporated water per area (g/m^2^/h) is calculated by measuring chronological water mass change in the petri dish. We have previously shown this artificial skin-based method [28] was able to provide controlled TEWL testbed for 9.23–76.91 (g/m^2^/h). For conductance measurement, we used resistors ranging from 0.5 to 5 MΩ, matching to the reported human skin resistance range [14], as a reference. In a voltage divider applied to this pen-type device, a fixed resistor of the PCB circuit plays a critical role to match the required measuring range to the skin, and thus provides proper output voltage range to the PCB. We selected a 5.5 MΩ-fixed resistor to get the optimal conductance range. For the hardness measurement, we prepared a series of silicone rubber blocks with varying elasticity. Polydimethylsiloxane (PDMS) (Sylgard 184, Dow Corning, Midland, MI, USA) was used with various mixing ratios of base and curing agent, ranging from 10:1 to 40:1. As the proportion of the curing agent decreases, the cured silicone rubber block softens [34]. The reference hardness of the silicone rubber blocks was determined by the standard ASTM D2240 using a Durometer type 00 (GS-754G, Teclock, Nagano, Japan). The average from five durometer measurements are used as the reference hardness of the silicone rubber bocks. The response to silicone blocks from the pen-type device are calibrated to the reference hardness. 

The performances for each property (TEWL, conductance, hardness) are evaluated in terms of sensitivity and linearity from the measured calibration curve. A linear regression using the least square method was used to calculate the sensors’ sensitivity and linearity that are derived from the stimulation-response curve.

## 3. Results and Discussion

**Figure 3:**
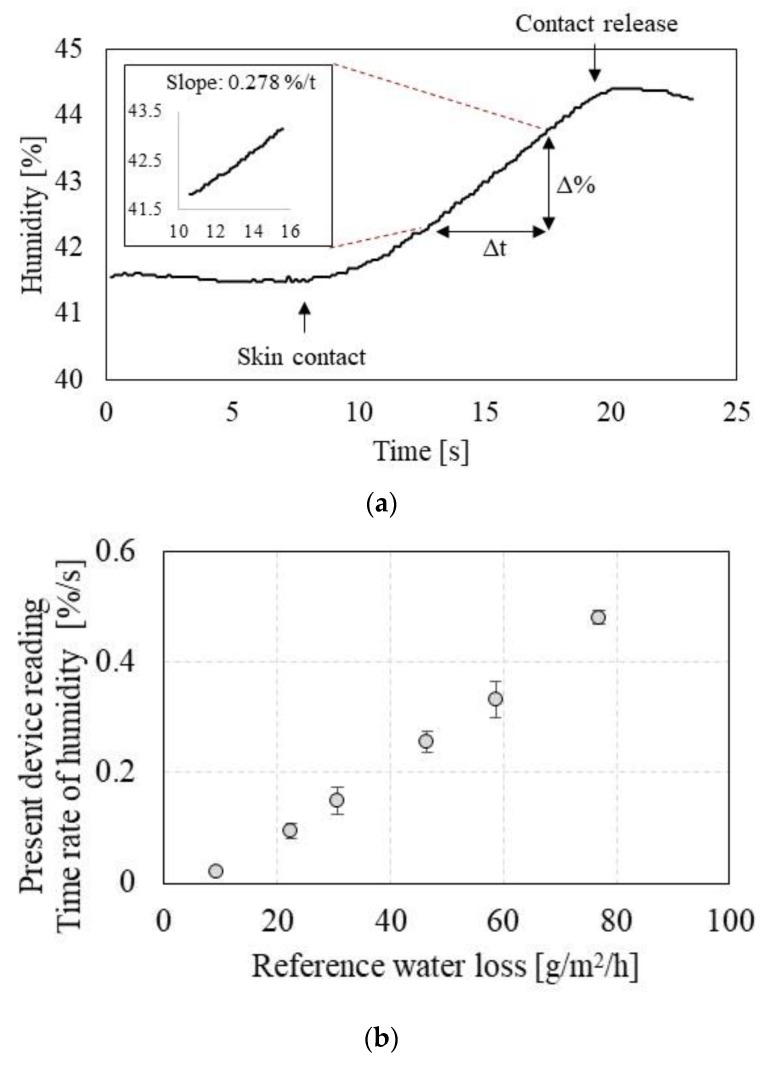
Transepidermal Water Loss (TEWL) measurement characterization, where the truncated hollow cone (THC) probe is in contact with the semipermeable membrane of the artificial skin: (**a**) the relative humidity profile over time; (**b**) calibration profile indicating the time rate of humidity depending on the reference water loss from the artificial skin.

Figure 3 shows TEWL measurement results from the pen-type device: Figure 3a shows humidity profile over time when the THC probe is in contact with a surface of the semipermeable membrane of the artificial skin. Humidity increases as the THC probe chamber collects the water for the first 10–15 s. A slope of the linear interval, the TEWL parameter, is shown as in inset in Figure 3a. Figure 3b shows the calibration results indicating the rate of humidity change (slope, %/s) emitted from the semipermeable membrane surface. The present pen-type device shows a sensitivity of 0.0068 (%/s)/(g/m^2^/h) with the high linearity of 99.63%. We also compared the measurement results with the commercial skin analysis device [27]. Figure S1 shows the TEWL measurement comparison between the commercial sensor and the present pen-type device. Coefficient of determination shows 0.8613, indicating a good agreement of a linear relation between two devices.

**Figure 4:**
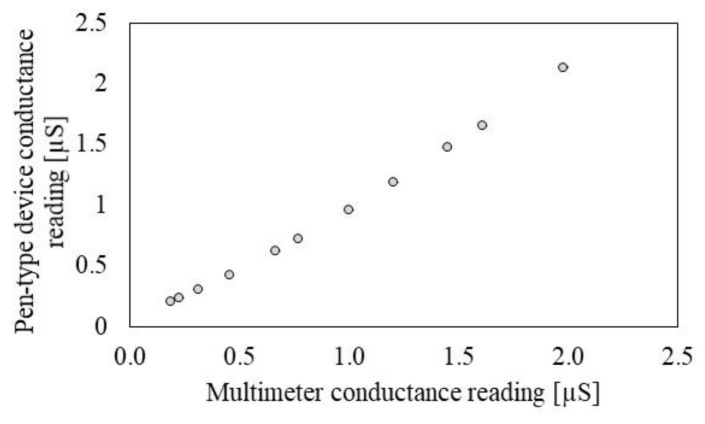
Conductance measurement performance of the present pen-type device for the reference resisters.

Figure S2 shows conductance measurements obtained from the raw data of the pen-type device. The pen-type device shows good conductance measurement capability in the range of 0.2–2.0 µS which is common human skin conductance range. The experimentally obtained calibration function is [*Output =* 994.73 *×* (*known conductance*)^−0.754^], and the coefficient of determination (*R*^2^) is 0.99. Figure 4 shows the calibrated conductance obtained from pen-type device as compared to the digital multimeter measurement. The pen-type device measures conductance with a sensitivity of 1.02 µS/µS and a linearity of 99.36%. 

**Figure 5:**
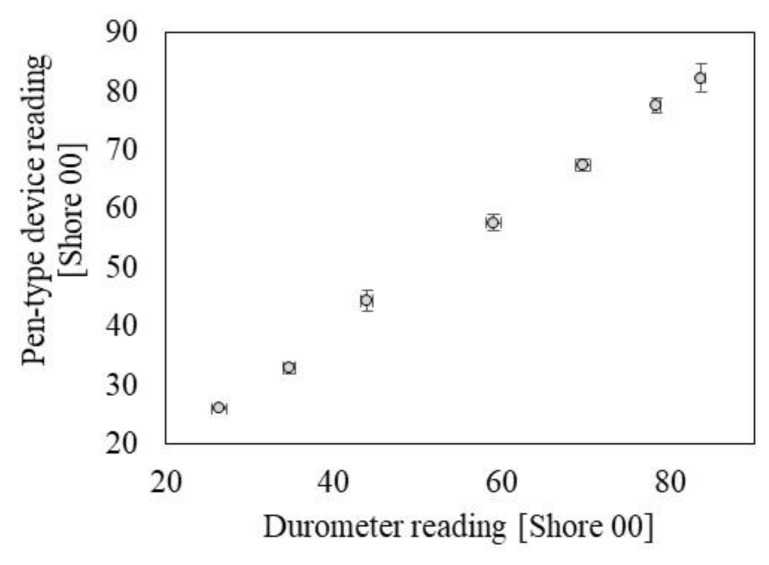
Hardness measurement performance of the pen-type device compared with durometer readings for the reference silicone rubber blocks.

Figure S3 shows the durometer outputs for the seven different PDMS cylindrical blocks where mixing ratio of the base and the curing agent varies from 40:1 to 10:1. The hardness of the seven blocks are measured as in the range of 26.4–83.7 shore 00. To obtain a calibration curve of the pen-type device’s hardness measurement, the load cell outputs are plotted over the reference durometer outputs. The hardness measurement performance varies when the wire-diameter (WD) of the mechanical coiled spring changes. Figure S4 indicates that the WD of 1.7 mm (Figure S4b) exhibits the optimal performances in terms of a sensitivity and a repeatability, as compared to those of the 1.6 and 1.8 mm WD devices (Figure S4a,c, respectively). Based on the Figure S4b, Figure 5 shows the calibrated hardness measurement performance compared with the durometer measurement. The pen-type device shows hardness measurement with a sensitivity of 0.98 Shore 00/Shore 00 and a linearity of 99.85% in the range of 26.4–83.7 shore 00. Based on the measurement performances characterizations, we experimentally prove that the present pen-type device has proper measurement capabilities for the detection of TEWL, skin conductance, and skin hardness in the range of human skin properties.

**Figure 6:**
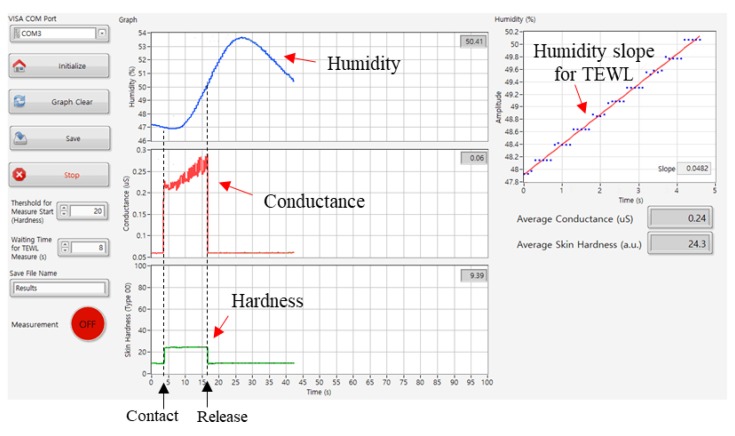
Demonstration of simultaneous measurement of the three different skin properties of TEWL, skin conductance, and skin hardness, in real-time.

Figure 6 shows the example of simultaneous measurement of the skin properties. Upon making contact to the artificial skin, humidity increment, conductance decrement, and hardness increment were clearly observed. These indicate that the pen-type device is a promising multimodal skin analysis tool without any crosstalk from the integrated sensors. While the pressure and conductance sensors are recovering into the dormant status instantaneously, the humidity sensor recovery requires time to the base line. Since we used the closed chamber principle (CCP) method [4,32] for the TEWL measurement, each measurement requires the ventilation time to recover to initial humidity level after the former measurement. The present pen-type device needs about 1 min ventilation time for each measurement to get repeatable slopes. Required ventilation time can be changed depending on the volume, length, and entrance diameter of the THC probe.

Measurement period of the present device is limited due to the initial time delay required for humidity increasing and recovery time for humidity recovery. Both are strongly related to the internal volume of THC and air ventilation. Small internal volume of THC is advantageous for both fast response and recovery. However, it decreases measurement range and sensitivity due to the limited humidity collection capability and decreased humidity slope. There is a trade-off between the response/recovery time and the sensitivity/range. Therefore, determination of the appropriate chamber size is important depending on the required application.

There exist other design considerations that affect sensor performance. The entrance hole diameter of the THC probe is especially important for the pen-type device design. For example, narrower entrance will show better sensitivity due to the deeper indentation on the skin. However, too narrow of an entrance can penetrate a skin surface, causing the invasive result and measurement error. Therefore, appropriate diameter of entrance hole is required for human skin hardness measurement. At the same time, it is also related to TEWL measurement. Smaller diameter of the entrance hole more lengthens the required humidity collection time, resulting in the longer measurement process for TEWL. Paired electrodes for skin conductance measurement are placed at the edge of the THC probe entrance. There can be technically difficult-to-place electrodes if the diameter of entrance hole is under 2 mm diameter due to the minimum size of the pin-electrode. Therefore, the present pen-type device was designed to have 3 mm for the entrance hole diameter, which is the proper dimensions for measuring the three skin properties.

The hardness measurement result is different depending on the contacting force. Therefore, it is important to keep the contacting force stable and consistent during the measurement period. Durometer, as the standard hardness measuring device, requires consistent contacting force [28] to obtain repeatable measurement results. We used stable contacting force of 8 N for this pen-type device to obtain repeatable measurement results.

The present pen-type device only considered the conductance for skin surface moisture assessment. In order to consider skin moisture characteristics underneath the skin epidermis, impedance analysis is required. By sweeping the AC frequency, a number of impedance measurements will provide skin moisture in deeper skin tissues.

The research scope of this study is to design, fabricate, and characterize the skin analyzing sensors based on the widely accepted calibration methods to evaluate each sensors performances. The limitation of this work is a clinical application test. For example, human skin measurements to analyze and evaluate human skin health states or physiological stress states from the pen-type device measurement can be one of the future work.

## 4. Conclusions

We presented the pen-type multimodal skin analysis device measuring TEWL, skin conductance, and skin hardness. The reported skin analysis device makes it possible to measure skin property conveniently. The present pen-type device, calibrated with artificial skins, showed appropriate measurement performances with good linearities in human ranges. Therefore, the present device can provide the users with portability and multimodality, enabling future applications, not only to the dermatology clinical/cosmetic practice, but also human performance measurements.

## Supplementary Materials

The following are available online at https://www.mdpi.com/1424-8220/19/18/3857/s1, Figure S1: TEWL measurement comparison between the commercial GPSkin device and the present multimodal device.; Figure S2: Conductance law data reading from the present pen-type device.; Figure S3: PDMS cylindrical blocks’ hardness measurement by the conventional durometer type 00 where PDMS’s mixing ratio of the base and the curing agent varies from 40:1 to 10:1.; Figure S4: Load cell reading depending on the hardness for the three different spring wire diameters of: (a) 1.6 mm; (b) 1.7 mm; and (c) 1.8 mm.
