# Supplementary material for: Portable Skin Analyzers with Simultaneous Measurements of Transepidermal Water Loss, Skin Conductance and Skin Hardness
Source: Sensors (Basel). 2019 Sep 6;19(18):3857. doi: 10.3390/s19183857 (PMC6767198; doi:10.3390/s19183857)
Supplement: Supplementary file 1 [file sensors-19-03857-s001.pdf]

< Supplementary materials >

Portable Skin analyzers with Simultaneous Measurements of Transepidermal Water Loss, Skin Conductance, and Skin Hardness

Daniel (Jai Kyoung) Sim[1,2], Sung Mok Kim[1], Steve S. Kim[2], and Il Doh[1,3,*]

[1] Center for Medical Convergence Metrology, Korea Research Institute of Standards and Science (KRISS), 267 Gajeong-ro, Yuseong-gu, Daejeon 34113, Republic of Korea

[2] 711th Human Performance Wing, Air Force Research Laboratory (AFRL), Wright-Patterson Air Force Base, Ohio 45433, United States

[3] Department of Medical Physics, University of Science and Technology (UST), 217 Gajeong-ro, Yuseong-gu, Daejeon 34113, Republic of Korea

[*] Corresponding author at: Korea Research Institute of Standards and Science (KRISS), Daejeon 34113, Republic of Korea

E-mail address: il.doh@kriss.re.kr (I. Doh)

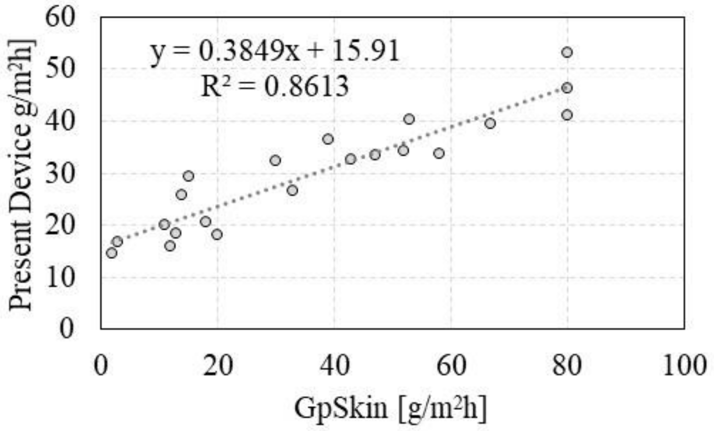

Figure S1. TEWL measurement comparison between the commercial GpSkin device and the present multimodal device.

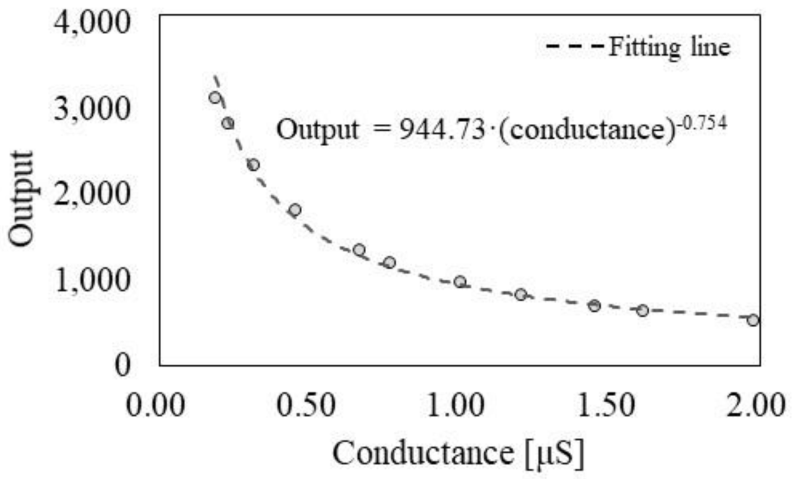

Figure S2. Conductance law data reading from the present pen-type device.

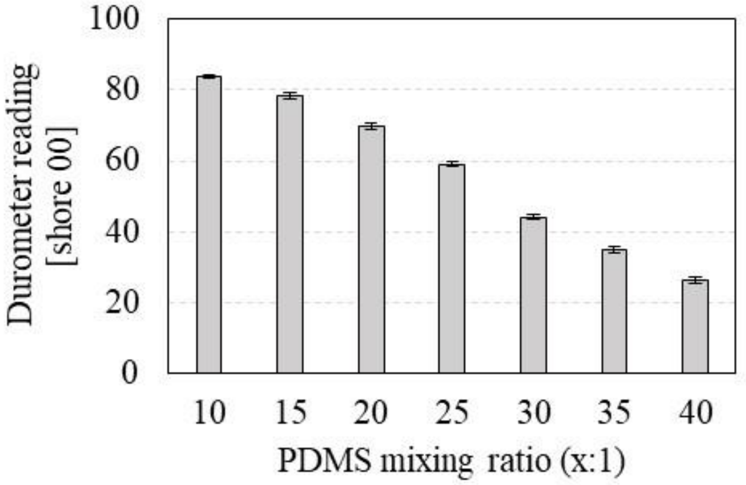

Figure S3. PDMS cylindrical blocks' hardness measurement by the conventional durometer type 00 where PDMS's mixing ratio of the base and the curing agent varies from 40:1 to 10:1.

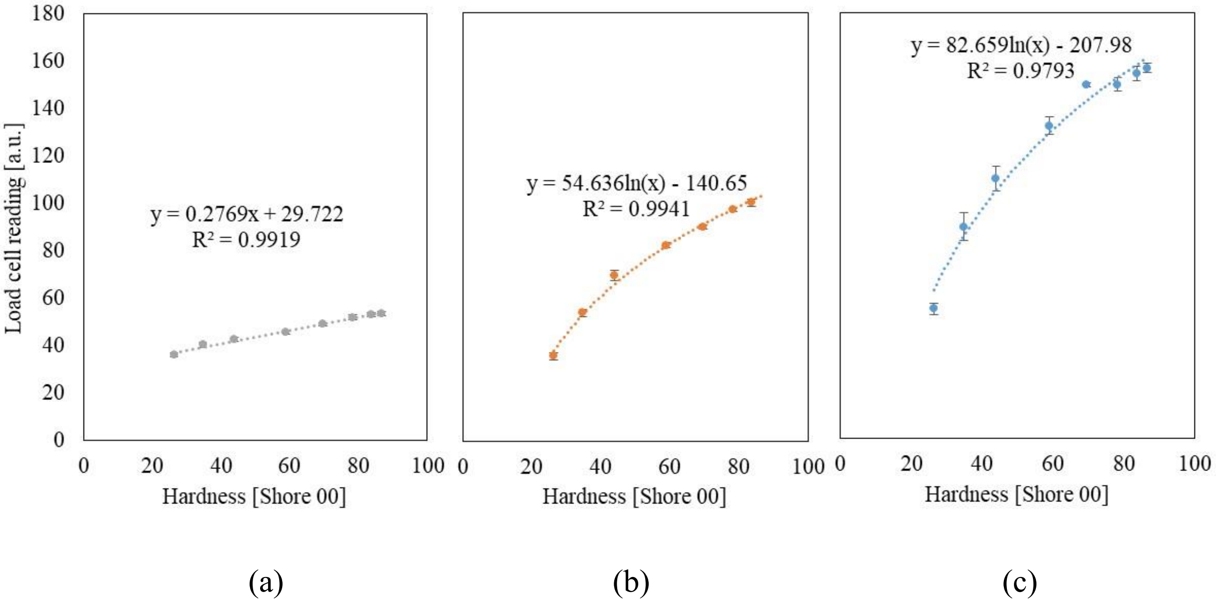

Figure S4. Load cell reading depending on the hardness for the three different spring wire diameters of: (a) 1.6 mm; (b) 1.7 mm; and (c) 1.8 mm.
